# Supplementary material for: Machine Learning Accelerates Crystallization for Structure Determination
Source: Angew Chem Int Ed Engl. 2026 May 4;65(25):e1218503. doi: 10.1002/anie.1218503 (PMC13266958; doi:10.1002/anie.1218503)

checkCIF/PLATON report

Structure factors have been supplied for datablock(s) lcz084

THIS REPORT IS FOR GUIDANCE ONLY. IF USED AS PART OF A REVIEW PROCEDURE FOR PUBLICATION, IT SHOULD NOT REPLACE THE EXPERTISE OF AN EXPERIENCED CRYSTALLOGRAPHIC REFEREE.

No syntax errors found. CIF dictionary Interpreting this report

Datablock: lcz084

Bond precision: C-C = 0.0081 A Wavelength=1.54184

Cell:	a=7.5537(1)	b=24.6709(2)	c=19.7226(2)
	alpha=90	beta=93.452(1)	gamma=90
Temperature:	100 K		

	Calculated	Reported
Volume	3668.77(7)	3668.77(7)
Space group	P 21/n	P 1 21/n 1
Hall group	-P 2yn	-P 2yn
Moiety formula	C22 H11 Ag3 F18 N6 S, C7 H8 S	C15 H3 Ag3 F17.992 N6, 2(C7 H8 S)
Sum formula	C29 H19 Ag3 F18 N6 S2	C29 H19 Ag3 F17.99 N6 S2
Mr	1181.08	1181.09
Dx,g cm-3	2.138	2.138
Z	4	4
Mu (mm-1)	14.954	14.954
F000	2279.7	2280.0
F000'	2292.55	
h,k,lmax	9,31,24	9,30,24
Nref	7844	7589
Tmin,Tmax	0.069,0.224	0.295,1.000
Tmin'	0.005	

Correction method= # Reported T Limits: Tmin=0.295 Tmax=1.000
AbsCorr = MULTI-SCAN

Data completeness= 0.967   Theta(max)= 77.855

R(reflections)= 0.0635(6762)   wR2(reflections)= 0.1748(7589)

S = 1.061   Npar= 526

The following ALERTS were generated. Each ALERT has the format
test-name_ALERT_alert-type_alert-level.
Click on the hyperlinks for more details of the test.

Alert level C

```
PLAT041_ALERT_1_C Calc. and Reported SumFormula    Strings  Differ     Please Check
              Calc: C29 H19 Ag3 F18 N6 S2
              Rep.: C29 H19 Ag3 F17.99 N6 S2
PLAT042_ALERT_1_C Calc. and Reported MoietyFormula Strings  Differ     Please Check
              Calc: C22 H11 Ag3 F18 N6 S, C7 H8 S
              Rep.: C15 H3 Ag3 F17.992 N6, 2(C7 H8 S)
PLAT112_ALERT_2_C ADDSYM Detects New (Pseudo) Symm. Elem         C         80 %Fit
PLAT213_ALERT_2_C Atom Ag03            has ADP max/min Ratio .....        3.1 prolat
PLAT213_ALERT_2_C Atom F00P            has ADP max/min Ratio .....        3.1 prolat
PLAT213_ALERT_2_C Atom F00T            has ADP max/min Ratio .....        3.9 prolat
PLAT213_ALERT_2_C Atom F013            has ADP max/min Ratio .....        3.8 prolat
PLAT220_ALERT_2_C NonSolvent   Resd 1  F   Ueq(max)/Ueq(min) Range        3.1 Ratio
PLAT241_ALERT_2_C High   'MainMol' Ueq as Compared to Neighbors of       Ag03 Check
PLAT342_ALERT_3_C Low Bond Precision on  C-C Bonds ...............    0.00813 Ang.
PLAT911_ALERT_3_C Missing FCF Refl Between Thmin & STh/L=    0.600         11 Report
                5  6  1,  -3 25  1,  -4  0  2,   0  0  2,  -2  0  4,  -1  0 11,
               -2 24 13,  -2 23 14,  -2 22 15,   5  6 18,  -4  0 20,
PLAT972_ALERT_2_C Check Calcd Resid. Dens.  0.73Ang From Ag01           -2.28 eA-3
PLAT972_ALERT_2_C Check Calcd Resid. Dens.  0.90Ang From Ag03           -2.25 eA-3
PLAT972_ALERT_2_C Check Calcd Resid. Dens.  0.87Ang From Ag02           -2.16 eA-3
PLAT972_ALERT_2_C Check Calcd Resid. Dens.  0.83Ang From Ag01           -2.14 eA-3
PLAT972_ALERT_2_C Check Calcd Resid. Dens.  0.81Ang From Ag02           -1.91 eA-3
PLAT972_ALERT_2_C Check Calcd Resid. Dens.  0.68Ang From Ag03           -1.82 eA-3
PLAT972_ALERT_2_C Check Calcd Resid. Dens.  0.72Ang From Ag03           -1.71 eA-3
PLAT972_ALERT_2_C Check Calcd Resid. Dens.  0.75Ang From Ag01           -1.67 eA-3
PLAT972_ALERT_2_C Check Calcd Resid. Dens.  0.71Ang From Ag02           -1.56 eA-3
```

Alert level G

```
PLAT003_ALERT_2_G Number of Uiso or U(i,j)  Restrained non-H-Atoms          1 Report
PLAT068_ALERT_1_G Reported F000 Differs from Calcd (or Missing)...     Please Check
PLAT072_ALERT_2_G SHELXL First  Parameter in WGHT  Unusually Large       0.13 Report
PLAT083_ALERT_2_G SHELXL Second Parameter in WGHT  Unusually Large       6.01 Why ?
PLAT142_ALERT_4_G s.u. on b - Axis Small or Missing .............    0.00020 Ang.
PLAT186_ALERT_4_G The CIF-Embedded .res File Contains ISOR Records          1 Report
PLAT232_ALERT_2_G Hirshfeld Test Diff (M-X)  Ag03     --S004      .      41.4 s.u.
PLAT242_ALERT_2_G Low    'MainMol' Ueq as Compared to Neighbors of       C018 Check
PLAT242_ALERT_2_G Low    'MainMol' Ueq as Compared to Neighbors of       C01B Check
PLAT242_ALERT_2_G Low    'MainMol' Ueq as Compared to Neighbors of       C01C Check
PLAT242_ALERT_2_G Low    'MainMol' Ueq as Compared to Neighbors of       C01J Check
PLAT242_ALERT_2_G Low    'MainMol' Ueq as Compared to Neighbors of       C01K Check
PLAT301_ALERT_3_G Main Residue  Disorder .............(Resd    1)          2% Note
```

```
PLAT720_ALERT_4_G Number of Unusual/Non-Standard Labels ..........         77 Note
              Ag01    Ag02    Ag03    S004    S005    F006    F007    F008
              F009    F00A    F00B    F00C    F00D    F00E    F00F    N00G
              N00H    N00I    N00J    N00K    N00L    F00M    F00N    F00O
              F00P    F00Q    C00R    H00R    C00S    H00S    F00T    C00U
              C00V    C00W    C00X    C00Y    H00Y    C00Z    C010    H010
              C011    C012    F013    C014    H014    C015    C016    F017
              C018    C019    H019    C01A    H01A    C01B    C01C    C01D
              H01E    C01E    H01F    C01F    H01G    C01G    H01H    C01H
              H01B    H01C    H01D    C01I    H01I    C01J    C01K    C01L
              H01J    H01K    H01L    C01M    H01M
PLAT860_ALERT_3_G Number of Least-Squares Restraints .............          6 Note
PLAT883_ALERT_1_G Absent Datum for _atom_sites_solution_primary ..     Please Do !
PLAT912_ALERT_4_G Missing # of FCF Reflections Above STh/L=  0.600        237 Note
PLAT913_ALERT_3_G Missing # of Very Strong Reflections in FCF ....          1 Note
               -2  0  4,
PLAT941_ALERT_3_G Average HKL Measurement Multiplicity ...........        3.3 Low
PLAT969_ALERT_5_G The 'Henn et al.' R-Factor-gap value ...........      3.141 Note
              Predicted wR2: Based on SigI**2  5.56 or SHELX Weight 16.48
PLAT978_ALERT_2_G Number C-C Bonds with Positive Residual Density.          0 Info
```

```
   0 ALERT level A = Most likely a serious problem - resolve or explain
   0 ALERT level B = A potentially serious problem, consider carefully
  20 ALERT level C = Check. Ensure it is not caused by an omission or oversight
  21 ALERT level G = General information/check it is not something unexpected

   4 ALERT type 1 CIF construction/syntax error, inconsistent or missing data
  26 ALERT type 2 Indicator that the structure model may be wrong or deficient
   6 ALERT type 3 Indicator that the structure quality may be low
   4 ALERT type 4 Improvement, methodology, query or suggestion
   1 ALERT type 5 Informative message, check
```

Validation response form

Please find below a validation response form (VRF) that can be filled in and pasted into your CIF.

```
# start Validation Reply Form
_vrf_PLAT041_lcz084
;
PROBLEM: Calc. and Reported SumFormula    Strings  Differ     Please Check
RESPONSE: ...
;
_vrf_PLAT042_lcz084
;
PROBLEM: Calc. and Reported MoietyFormula Strings  Differ     Please Check
RESPONSE: ...
;
_vrf_PLAT112_lcz084
;
PROBLEM: ADDSYM Detects New (Pseudo) Symm. Elem         C         80 %Fit
RESPONSE: ...
;
_vrf_PLAT213_lcz084
;
PROBLEM: Atom Ag03            has ADP max/min Ratio .....        3.1 prolat
```

```
RESPONSE: ...
;
_vrf_PLAT220_lcz084
;
PROBLEM: NonSolvent   Resd 1  F   Ueq(max)/Ueq(min) Range        3.1 Ratio
RESPONSE: ...
;
_vrf_PLAT241_lcz084
;
PROBLEM: High   'MainMol' Ueq as Compared to Neighbors of       Ag03 Check
RESPONSE: ...
;
_vrf_PLAT342_lcz084
;
PROBLEM: Low Bond Precision on  C-C Bonds ...............    0.00813 Ang.
RESPONSE: ...
;
_vrf_PLAT911_lcz084
;
PROBLEM: Missing FCF Refl Between Thmin & STh/L=    0.600         11 Report
RESPONSE: ...
;
_vrf_PLAT972_lcz084
;
PROBLEM: Check Calcd Resid. Dens.  0.73Ang From Ag01           -2.28 eA-3
RESPONSE: ...
;
# end Validation Reply Form
```

It is advisable to attempt to resolve as many as possible of the alerts in all categories. Often the minor alerts point to easily fixed oversights, errors and omissions in your CIF or refinement strategy, so attention to these fine details can be worthwhile. It is up to the individual to critically assess their own results and, if necessary, seek expert advice.

PLATON version of 17/09/2025; check.def file version of 17/09/2025

---

duplicate check

No duplication found

Datablock lcz084 - ellipsoid plot

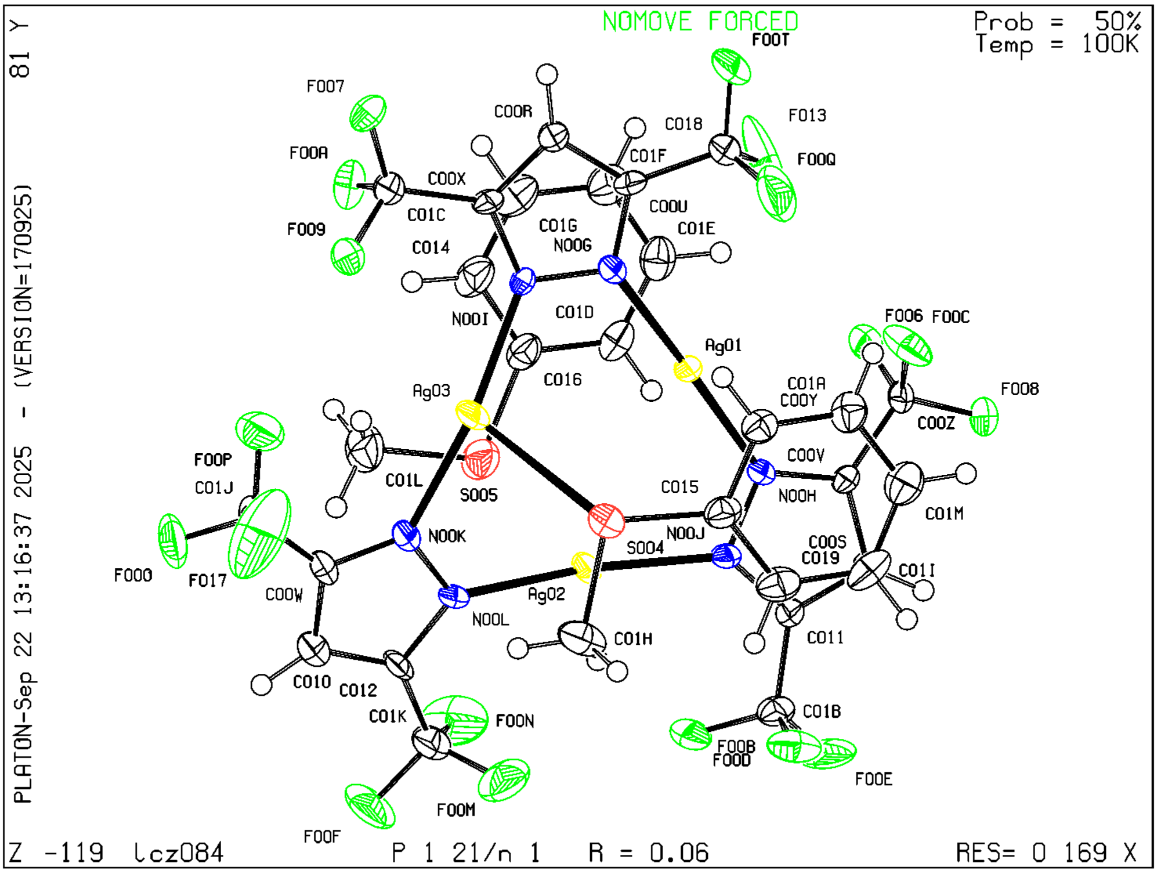

Supplement: Supplementary file 2 — Supporting File 2: anie72462‐sup‐0002‐DataFile.zip. [file ANIE-65-e1218503-s002.zip › supplementary_materials_crystallographic_data submit.zip!/cif_and_checkcif(1-114)/Ag3Pz3_84/Ag3Pz3_84_checkcif.pdf]
